# Increased accumulation of hypoxia-inducible factor-1α with reduced transcriptional activity mediates the antitumor effect of triptolide

**DOI:** 10.1186/1476-4598-9-268

**Published:** 2010-10-11

**Authors:** Zhao-Li Zhou, Zhi-Guo Luo, Bing Yu, Yi Jiang, Yi Chen, Jian-Ming Feng, Mei Dai, Lin-Jiang Tong, Zheng Li, Yuan-Chao Li, Jian Ding, Ze-Hong Miao

**Affiliations:** 1 Division of Antitumor Pharmacology, State Key Laboratory of Drug Research, Shanghai Institute of Materia Medica, Chinese Academy of Sciences, Shanghai 201203, China; 2 Department of Medicinal Chemistry, State Key Laboratory of Drug Research, Shanghai Institute of Materia Medica, Chinese Academy of Sciences, Shanghai 201203, China; 3 Department of Medical Oncology, Cancer Hospital, Shanghai Medical School, Fudan University, Shanghai 200032, China; 4 Department of Pharmacology, Shenyang Pharmaceutical University, 103 Wenhua Road, Shenhe District, Shenyang 110016, China

## Abstract

**Background:**

Hypoxia-inducible factor-1α (HIF-1α), a critical transcription factor to reduced O_2 _availability, has been demonstrated to be extensively involved in tumor survival, aggressive progression, drug resistance and angiogenesis. Thus it has been considered as a potential anticancer target. Triptolide is the main principle responsible for the biological activities of the Traditional Chinese Medicine *tripterygium wilfordii *Hook F. Triptolide possesses great chemotherapy potential for cancer with its broad-spectrum anticancer, antiangiogenesis, and drug-resistance circumvention activities. Numerous biological molecules inhibited by triptolide have been viewed as its possible targets. However, the anticancer action mechanisms of triptolide remains to be further investigated. Here we used human ovarian SKOV-3 cancer cells as a model to probe the effect of triptolide on HIF-1α.

**Results:**

Triptolide was observed to inhibit the proliferation of SKOV-3 cells, and meanwhile, to enhance the accumulation of HIF-1α protein in SKOV-3, A549 and DU145 cells under different conditions. Triptolide did not change the kinetics or nuclear localization of HIF-1α protein or the 26 S proteasome activity in SKOV-3 cells. However, triptolide was found to increase the levels of HIF-1α mRNA. Unexpectedly, the HIF-1α protein induced by triptolide appeared to lose its transcriptional activity, as evidenced by the decreased mRNA levels of its target genes including VEGF, BNIP3 and CAIX. The results were further strengthened by the lowered secretion of VEGF protein, the reduced sprout outgrowth from the rat aorta rings and the inhibitory expression of the hypoxia responsive element-driven luciferase reporter gene. Moreover, the silencing of HIF-1α partially prevented the cytotoxicity and apoptosis triggered by triptolide.

**Conclusions:**

The potent induction of HIF-1α protein involved in its cytotoxicity, together with the suppression of HIF-1 transcriptional activity, indicates the great therapeutic potential of triptolide as an anticancer drug. Meanwhile, our data further stress the possibility that HIF-1α functions in an unresolved nature or condition.

## Background

Hypoxia-inducible factor-1α (HIF-1α) is a critical transcription factor responsible for adaptive responses of cancer cells to reduced O_2 _availability [1]. Through modulation of the expression of at least 70 genes, HIF-1α is extensively involved in tumor survival, aggressive progression, drug resistance and angiogenesis [2]. Elevated levels of HIF-1α protein are observed in various human primary and metastatic cancers, either as direct results of intratumoral hypoxia or secondary to genetic alterations in oncogenes or tumor suppressor genes [3,4]. Those cancers are generally relatively poorly responsive to chemotherapy or radiotherapy with poor prognosis [5]. Thus, HIF-1α has been proposed as a promising anticancer target [6]. On the other hand, HIF-1α(-/-) tumors have also been demonstrated to show a feature of faster proliferation and more obvious resistance to apoptosis than the HIF-1α(+/+) counterparts [7], suggesting a possibility that HIF-1α may have unknown function(s) or exert its transcriptional activity dependent on specific, undefined conditions or stimulations.

Triptolide is an effective principle of the Traditional Chinese Medicine *tripterygium wilfordii *Hook F that has been used to treat autoimmune and inflammatory diseases for centuries [8,9]. Triptolide possesses broad-spectrum anticancer, antiangiogenesis, and drug-resistance circumvention activities [10-13]. Moreover, our recent study shows that a novel C14-hydroxyl substituted triptolide derivative elicits selective anticancer effects, specifically against ovarian and prostate cancers in nude mice xenograft models, with reduced toxicity as compared to the parent triptolide [14]. Nevertheless, the anticancer action mechanisms of triptolide are complicated and remain to be further investigated. Triptolide downregulates various proteins including heat shock protein 70, Bcr-Abl, survivin, Mcl-1, Akt, c-myc, cyclin A/cdk2, cyclin B/cdc2, cyclin D1 and pRB, which may contribute to its anticancer activity under specific conditions [12,15,16]. In addition, inhibition of nuclear factor κB activation by triptolide is also assoctiated with its potentiation of TNF related apoptosis-inducing ligand-induced anticancer effects [17]. Notably, however, triptolide enhances the levels of p53 protein in p53-wild type human tumors but lowers its transcriptional activity, resulting in the reduced expression of p21 protein [18-20].

Based on the discovery of selective anticancer activity of the new triptolide analogue in human ovarian and prostate cancer xenograft models [14], in this current study, we used human ovarian SKOV-3 cancer cells as a model to further investigate the mechanisms of action of triptolide. Triptolide was unexpectedly revealed to enhance the cellular accumulation of HIF-1α protein with reduced transcriptional activity. The impact of triptolide on HIF-1α contributes to its partial anticancer effect. These results from triptolide further stress the possibility that HIF-1α functions in an unresolved nature or condition.

## Results

### Triptolide elicits cytotoxicity and increases HIF-1α accumulation

Triptolide has been demonstrated to possess potent antitumor and antiangiogenic activities [12,21]. HIF-1α is an important regulator of tumor angiogenesis [22]. To find a proper model to examine the effect of triptolide on HIF-1α protein, we first tested the sensitivity of human ovarian cancer SKOV-3 cells to this compound because the cells are highly sensitive, both *in vitro *and *in vivo*, to triptolide analogues [14].

**Figure 1:**
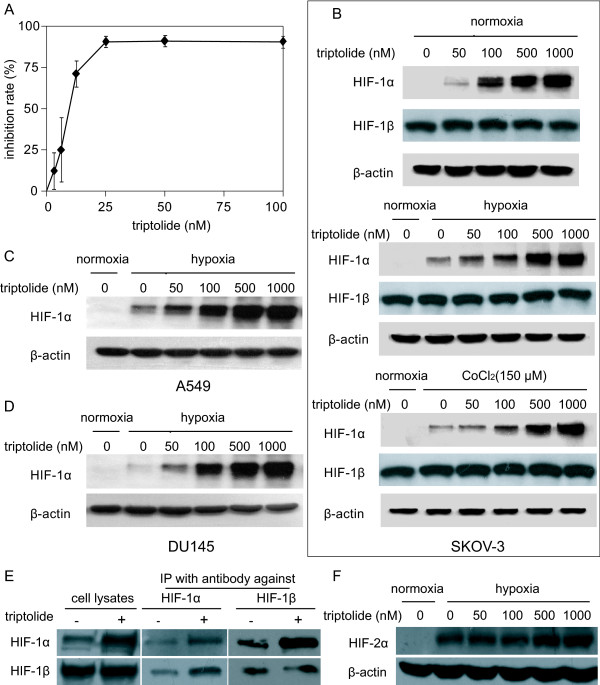
**Triptolide inhibited the proliferation of SKOV-3 cells and induced the accumulation of HIF-1α protein**. ***A***. SKOV-3 cells were exposed to triptolide of gradient concentrations for 72 h. The proliferation inhibition was determined by SRB assays. ***B***. SKOV-3 cells at normoxia (upper panel), 1% O_2 _hypoxia (middle panel) or CoCl_2 _(150 μM) mimic hypoxia (lower panel) were treated with tiptolide for 12 h and then subjected to standard Western blotting analyses for the levels of HIF-1α and HIF-1β proteins. ***C ***and ***D***. A549 (*C*) and DU145 (*D*) cells at 1% O_2 _hypoxia were treated with tiptolide for 12 h and Western blotting analyses were done as in ***B***. ***E***. SKOV-3 cells at CoCl_2 _(150 μM) mimic hypoxia were treated with tiptolide at 1000 nM for 12 h. Then the cells were subjected to Western blotting for the levels of HIF-1α, HIF-1β proteins (left panel); or the cells were used to do co-immunoprecipitation assays for the binding between HIF-1α and HIF-1β (middle and right panels). ***F***. SKOV-3 cells at 1% O_2 _hypoxia were treated with tiptolide at gradient concentrations for 12 h. Then the cells were subjected to Western blotting for the levels of HIF-2α proteins. All the experiments were performed three times and the representative results were presented.

As expected, triptolide elicited potent concentration-dependent cytotoxicity in SKOV-3 cells with an averaged IC_50 _value of 10.24 nM for 72-h treatments (Fig. 1A). Unexpectedly, however, triptolide led to significant accumulation of cellular HIF-1α protein in a concentration-dependent manner following the 12-h exposure of SKOV-3 cells at normoxia (Fig. 1B). The similar results were reproducible in SKOV-3 cells exposed to hypoxia or mimic hypoxia with cobalt chloride (CoCl_2_) [23] (Fig. 1B) or in hypoxic human lung A549 and prostate DU145 cancer cells (Fig. 1C and 1D). The data indicate that the increase in the cellular accumulation of HIF-1α protein by triptolide is independent of the environmental oxygen pressure and the cell type, suggesting that it is an inherent capability of this agent.

To function as a transcription factor, HIF-1α heterodimerizes HIF-1β that is constitutively expressed [2]. The treatment with triptolide did not change the levels of HIF-1β in normoxic, hypoxic or CoCl_2_-treated SKOV-3 cells (Fig. 1B). Moreover, co-immunoprecipitation further showed that triptolide did not impair the binding of HIF-1α to HIF-1β (Fig. 1E). In addition, triptolide just marginally changed the levels of HIF-2α (Fig. 1F), another hypoxia-inducible factor (HIF) alpha subunit that has various overlapped targeting genes with HIF-1α [24].

### Triptolide increases HIF-1α accumulation but does not change its kinetics and cellular localization at hypoxia

**Figure 2:**
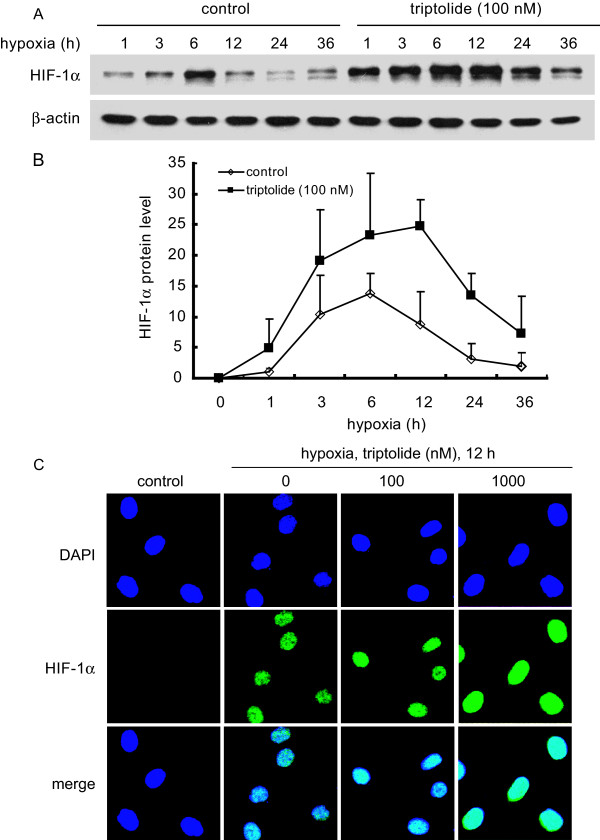
**Triptolide did not change the cellular kinetics and nuclear localization of HIF-1α protein in response to hypoxia**. ***A ***and ***B***. SKOV-3 cells were exposed to 1% O_2 _hypoxia in the presence or absence of triptolide (100 nM) for the indicated time periods. Then standard western blotting analyses were performed for the levels of HIF-1α protein (A). Adobe Photoshop CS2 version 9.0.2 was used for relative semi-quantification of the HIF-1α band intensity, which was normalized with β-Actin as the internal control. The results from three separate experiments were expressed as mean ± SD (B). ***C***. SKOV-3 cells were treated as described in the Materials and Methods section and then subjected to immunofluorescence analyses for the localization of HIF-1α protein. The representative images were from three separate experiments with similar results.

In response to acute hypoxia, HIF-1α protein rapidly accumulates in the cells due to the inactivation of oxygen-sensitive prolyl hydroxylase [25]; but prolonged hypoxia initiates CHIP (a prolyl hydroxylase -independent E3 ligase) -mediated HIF-1α degradation and lowers its cellular levels [26]. This typical first-up-and-then-down kinetics of HIF-1α protein also occurred in SKOV-3 cells at hypoxia (Fig. 2A and 2B). To clarify whether triptolide affects such kinetics, we exposed hypoxic SKOV-3 cells to this compound. The result showed that triptolide, though increasing the amount at each corresponding time-point, did not change the kinetic trend of the accumulation and degradation of HIF-1α protein (Fig. 2A and 2B). Moreover, HIF-1α protein was also localized in the nuclei of the hypoxic SKOV-3 cells treated with triptolide (Fig. 2C), just as generally expected [27,28]. These data critically suggest that triptolide may not interfere with HIF-1α protein degradation, either oxygen-dependent or oxygen-independent, when collectively considering the increased levels of HIF-1α protein at normoxia (Fig. 1B).

### Triptolide does not affect the 26 S proteasome activity but enhances the levels of HIF-1α mRNA

**Figure 3:**
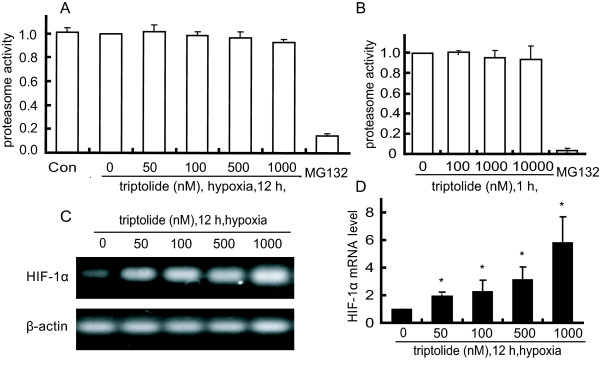
**Triptolide did not affect the 26 S proteasome activity but enhanced the levels of HIF-1α mRNA**. ***A***. SKOV-3 cells were treated with triptolide for 12 h under hypoxia condition and then harvested for the detection of 26 S proteasome activity as described in the Materials and Methods. ***B***. The lysates of SKOV-3 cells were incubated in the presence or absence of triptolide and then assayed for the 26 S proteasome activity. ***C ***and ***D***. The levels of HIF-1α mRNA from reverse transcription -PCR (C) and real-time PCR (D) analyses in the hypoxic SKOV-3 cells treated with triptolide for 12 h. The levels of HIF-1α mRNA were normalized with β-actin mRNA expression; columns, mean of three independent experiments; bars, SD. The significant difference between triptolide-treated groups and hypoxia-control groups was analyzed by Student t test. * P < 0.05.

To validate the effect of triptolide on HIF-1α protein degradation, we detected whether triptolide inhibited the activity of 26 S proteasome, which is crucial degradation machinery for HIF-1α protein [29]. Triptolide was not shown to apparently inhibit the chymotrypsin-like activity of 26 S proteasome in either SKOV-3 cells or their cell lysates treated with triptolide, even up to 10000 nM. In contrast, the positive control MG132 dramatically inhibited the 26 S proteasome activity (Fig. 3A and 3B).

To further dissect the possible cause of HIF-1α induction by triptolide, we used semi-quantitative RT-PCR and quantitative real-time PCR assays for the levels of HIF-1α mRNA in the triptolide-treated SKOV-3 cells. The results showed that triptolide enhanced the levels of HIF-1α mRNA in a concentration-dependent manner (Fig. 3C and 3D), which may be responsible for the HIF-1α accumulation.

### Triptolide reduces the transcriptional activity of HIF-1α protein

**Figure 4:**
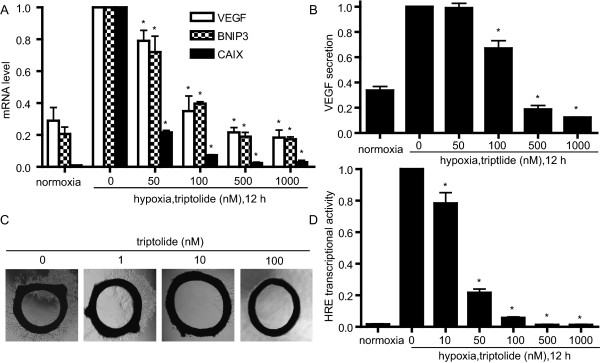
**Triptolide decreased the transcription activity of HIF-1α protein**. ***A***. SKOV-3 cells were cultured under normoxic or hypoxic conditions in the presence or absence of triptolide for 12 h. The mRNA levels of VEGF, BNIP3 and CAIX were analyzed by real-time PCR and normalized with β-actin mRNA expression. ***B***. ELISA assays were done for VEGF secretion from the SKOV-3 cells treated as in A. ***C***. Triptolide inhibited new microvessel outgrowth arising from rat aorta rings. The representative images were from three separate experiments with similar results. ***D***. MCF-7 cells were transiently transfected with the HRE-luciferase and renilla-luciferase reporter plasmids and then cultured at normoxia or hypoxia in the presence or absence of triptolide for 12 h followed by assays for luciferase activity. Data shown in A, B and D were expressed as mean ± SD, n = 3. The significant difference between triptolide-treated groups and hypoxia-control groups was analyzed by Student t test. * P < 0.05.

HIF-1α protein functions as a critical transcription factor in adaptive response to hypoxia [1]. To determine whether triptolide also increases the transcriptional activity of HIF-1α protein when enhancing its accumulation, we examined the expression levels of its several target genes including vascular endothelial growth factor (VEGF), BCL2 and adenovirus E1B 19-kDa-interacting protein 3 (BNIP3) and carbonic anhydrase IX (CAIX) [30,31] in the triptolide-treated SKOV-3 cells. Unexpectedly, however, the results revealed that the mRNA levels of the three genes did not increase but decreased typically in a concentration-dependent manner (Fig. 4A). Moreover, the secretion of VEGF protein, a critical angiogenesis factor, also reduced (Fig. 4B). Triptolide was further revealed to obviously inhibit the sprout outgrowth from the rat aorta rings (Fig. 4C), indicating its antiangiogenesis capability as previously reported [10,11,21].

To confirm whether triptolide reduces the transcriptional activity of HIF-1α protein, we used the hypoxia responsive element (HRE)-driven luciferase reporter gene assays. After failure with SKOV-3 or A549 cells due to the low transfection efficiency, we co-transfected the HRE luciferase reporter plasmid and the renilla luciferase reporter vector pGL-3 into MCF-7 cells for 24 h. Then the cells were treated with triptolide for additional 12 h. Triptolide reduced the luciferase-elicited fluorescence in a concentration-dependent fashion, and at 1000 nM of triptolide, the fluorescence almost lowered to the basal level (Fig. 4D). Collectively, the above evidence arising from all the levels of the transcription of the target genes, the reporter gene and the subsequent biological effects indicates that triptolide, though increasing the cellular accumulation of HIF-1α protein, reduces its transcriptional function.

### The action of triptolide on HIF-1α contributes at least partially to its anticancer activity

**Figure 5:**
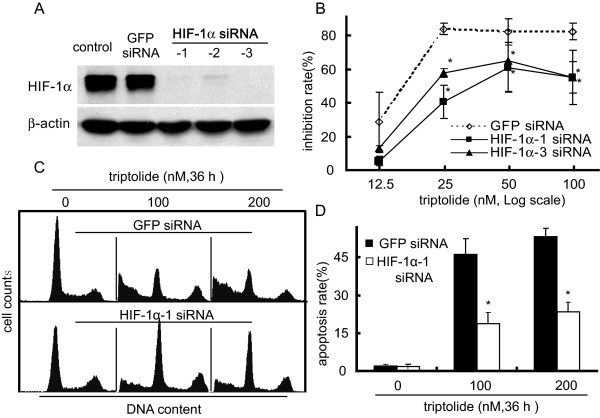
**HIF-1α silencing partially prevented the proliferation inhibition and apoptosis induction by triptolide**. ***A***. siRNAs targeting three different sequences of the HIF-1α gene were used to effectively silence this gene in SKOV-3 cells (Western blotting). GFP siRNA was used as scrambled control. ***B***. The SKOV-3 cells transfected with HIF-1α or GFP siRNA were treated with triptolide for 48 h and then subjected to SRB assays for the proliferation inhibition. ***C***. The SKOV-3 cells transfected with HIF-1α or GFP siRNA were treated with triptolide for 36 h and then assayed for apoptotic induction by flow cytometry as described in the Materials and Methods section. The representative histograms were from three independent experiments with similar results. ***D***. The apoptosis rate from C. Data shown in B and D were expressed as mean ± SD, n = 3. The significant difference between triptolide-treated groups and hypoxia-control groups was analyzed by Student t test. * P < 0.05.

To demonstrate whether the effect of triptolide on HIF-1α is associated with its anticancer activity, we knocked down HIF-1α with three specific HIF-1α siRNAs in SKOV-3 cells (Fig. 5A). SRB assays showed that the silencing of HIF-1α partially prevented the cytotoxicity of triptolide (Fig. 5B). Consistently, triptolide induced much less apoptosis in the HIF-1α-silenced cells than in the control cells (Fig. 5C and 5D). These data clearly reveal a close association of the effect of triptolide on HIF-1α with its anticancer activity.

## Discussion

Overexpression of HIF-1α in human cancers is usually correlated with poor prognosis and poor therapeutic response [32]. HIF-1α has been considered as a potential therapeutic target [33]. Nevertheless, there are also reports that HIF-1α null tumors grow unexpectedly fast, and that tumor cells with HIF-1α(-/-) tumors are more proliferative and less apoptotic than those with HIF-1α(+/+) even when tumor vessel formation was impaired [7]. These paradoxical observations seem to suggest that the roles of HIF-1α in tumor development and/or in cancer therapy are conditional and that the conditions remain to be further clarified so that this important tumor-related factor can be better employed. Our findings in this current study further stress such conditional or stimulation-dependnent changes as the potent anticancer agent triptolide has been revealed to increase the cellular accumulation of HIF-1α protein that contributes partially to its anticancer activity.

Triptolide has been well documented to possess potent, broad-spectrum antitumor activity and to inhibit angiogenesis [10,34]. Our present study showed that triptolide enhanced the cellular accumulation of HIF-1α protein in SKOV-3, A549 and DU145 cancer cells at normoxia, hypoxia or CoCl_2_-mimic hypoxia. However, triptolide did not apparently change the protein levels of HIF-1β and HIF-2α and the binding of HIF-1α to HIF-1β. Triptolide did not change the kinetics or localization of HIF-1α protein in SKOV-3 cells exposed to hypoxia. Consistently, triptolide did not impair the 26 S proteasome activity. The increased levels of HIF-1α mRNA could be responsible for the accumulation of HIF-1α protein induced by triptolide in SKOV-3 cells. Unexpectedly, the triptolide-increased HIF-1α protein seems to lose its transcriptional activity, as evidenced by the decreased mRNA levels of its target genes including VEGF, BNIP3 and CAIX (the transcription of which is generally promoted by HIF-1α [30,31]). The results were further strengthened by the lowered secretion of VEGF protein, the reduced sprout outgrowth from the rat aorta rings and the inhibitory expression of the HRE luciferase reporter gene. More importantly, the partial prevention of cytotoxicity and apoptotic induction of triptolide by HIF-1α silencing indicates the contribution of the accumulated HIF-1α protein to the anticancer action of this compound.

Several important points arise from those results: (1) The apparent association of the increased HIF-1α protein induced by triptolide with its anticancer activity challenges the general notion that the downregulation of HIF-1α protein elicits anticancer and antiangiogenic effects, as many HIF-1α inhibitors do [35,36]. (2) The HIF-1α protein in the triptolide-treated cells appears to have some additional non-transcriptional function because it loses its transcriptional activity but is indeed involved in the anticancer and antiangiogenic actvity of triptolide in our case. Another possibility is that the apparently decreased transcriptional activity of the triptolide-induced HIF-1α protein may be subsequent to the inhibitory effect of triptolide on RNA polymerase II (RNA Pol II). As a critical general transcription factor, RNA Pol II has been demonstrated to be inhibited by triptolide, which could contribute to its global transcriptional arrest [37,38]. The inhibition of RNA Pol II impairs the transcription-promoting function of HIF-1, thus reducing the transcriptional activity of the triptolide-induced HIF-1α protein. Actually, triptolide has been reported to similarly lead to the elevated levels of wild-type p53 protein with reduced transcriptional activity [18-20]. (3) The enhancement of HIF-1α mRNA by triptolide may be a compensatory response by the tumor cells in an effort to maintain HIF-1α transcriptional activity. Or, subsequent to its RNA Pol II inhibition, the transcription of some factor(s) responsible for degrading HIF-1α mRNA may be arrested, finally disrupting the degradation machinery and resulting in the accumulation of HIF-1α mRNA. This is potentially similar to the effect of actinomycin D, an inhibitor of transcription, on the degradation of HIF-1α protein. Actinomycin D was found to lead to super-induction of HIF-1α protein by inhibiting the transcription of some unidentified factor(s) responsible for degrading HIF-1α protein (transcription-dependent degradation) [39]. (4) Triptolide could be used as a tool compound to probe the potential new function of HIF-1α protein due to its unique action. (5) The interference of triptolide with HIF-1α is one of its possible anticancer and/or antiangiogenesis mechanisms.

Those points obviously deserve further investigation. Hypoxia is prevalent in solid tumors and HIF-1α is a critical regulator of tumor hypoxia adaption [40]. Clarification of the conditions or stimulations required for specific HIF-1α activities (for instance, promoting or suppressing the expression of specific genes or gene sets) may greatly help the development of HIF-1α-targeted therapeutics and the monitor of cancer progression and prognosis. Moreover, demonstration of the function(s) of HIF-1α in addition to its transcriptional activity, if any indeed as shown in this study, will give new insights into hypoxia biology. On the other hand, triptolide is a promising anticancer lead compound and its chemical modifications are actively ongoing [14,41]. The discovery of its unique impact on HIF-1α suggests another possible anticancer mechanism. Therefore, the questions of how triptolide increases the levels of HIF-1α mRNA, why and how it reduces the transcriptional activity of HIF-1α protein need thoroughly elucidating.

## Conclusions

In summary, triptolide was found to increase the levels of HIF-1α mRNA and protein and to reduce HIF-1α transcriptional activity, which contributes to its antitumor effect partially. These data, on one hand, propose a new potential anticancer mechanism of triptolide, and stress a possibility that HIF-1α functions in an unresolved nature or condition on the other.

## Methods

### Chemicals

Triptolide used in our experiments was prepared from *tripterygium wilfordii *Hook F with the following procedure. First, the powder of *tripterygium wilfordii *bark was extracted with ethanol to obtain its ethanol extracts. The ethanol extracts were next extracted with chloroform to get chloroform extracts. Then, we purified the chloroform extracts on silica gel columns with a mobile phase of chloroform to get triptolide crude. Finally, by recrystalizing the triptolide crude with ether, we obtained triptolide with the HPLC purity of more than 99%. MG132 was purchased from Sigma (St. Louise, MO, USA). All the compounds were dissolved at 10 mM in dimethyl sulfoxide (Sigma, St. Louise, MO, USA) as stock solution. The stock solutions were kept in aliquot at -20°C (triptolide) or at -80°C (MG132) and thawed immediately prior to each experiment.

### Cell culture

Human ovarian SKOV-3, lung A549, breast MCF-7 and prostate DU145 cancer cells were obtained from the American Type Culture Collection (Manassas, VA, USA). Cells were cultured in DMEM (SKOV-3 and MCF-7), F12 (A549), or RPIM-1640 medium (DU145) (Life Technologies, Grand Island, NY USA) supplemented with 15% FBS (Life Technologies, Grand Island, NY, USA) at 37°C in a humidified atmosphere containing 5% CO_2_. Hypoxia treatment was performed by placing cells in a CO_2 _Water Jacketed Incubator (Thermo Forma, Model 3110 series, OH, USA) flushed with a mixture of 1% O_2_, 5% CO_2 _and 94% N_2_.

### Cytotoxicity assays

The cytotoxicity of triptolide was examined by sulforhodamine B (SRB) assays as described previously [42]. Briefly, cells in 96-well plates were treated in triplicate with gradient concentrations of triptolide at 37°C for 72 h, and then assessed with SRB (Sigma, St. Louise, MO, USA). The absorbance at 560 nM was detected with a plate reader (SpectraMax, Molecular Devices, CA, USA). The growth inhibition rate was calculated as (1-A_560 treated_/A_560 control_) × 100%.

### Western blotting analyses

Cells were lyzed in 1 × SDS lysis buffer [50 mM Tris-HCl (pH6.8), 100 mM DTT, 2% SDS, 0.1% bromphenol blue, 10% glycerol] and then boiled for 10-15 min. Western blotting analyses were performed as previously described [43] using appropriate antibodies including anti-HIF-1α (BD, Franklin Lakes, NJ, USA), anti-HIF-1β (BD, Franklin Lakes, NJ, USA), anti-HIF-2α (R&D Systems, MN, USA), anti-β-actin (Beyotime, Haimen, China), and the levels of cellular proteins were visualized with peroxidase-coupled secondary antibodies (Dingguo, Beijing, China) using ECL-plus kit from Amersham Biosciences (Buckinghamshire, UK).

### Cell immunofluorescence

Immunofluorescence analyses were performed as described previously [27]. Briefly, Cells (0.5 × 10^5^) were seeded onto cover slips in 24-well plates and exposed to hypoxia for 12 h treated with or without triptolide simultaneously. One slip was left at normoxia as control. Then, the cells were fixed, washed, permeabilized, washed and blocked. After that, the cells were sequentially incubated with anti-HIF-1α antibody (BD, Franklin Lakes, NJ), washed, and incubated with Alexa Fluor 488-conjugated anti-mouse secondary antibody (Invitrogen, Carlsbad, CA). Finally, the cover slips were photographed with a Leica TCS SP2 confocal microscope (Leica, Wetzlar, Germany).

### Co-immunoprecipitation

Cells treated with 150 μM CoCl_2 _with or without 1000 nM triptolide for 12 h were collected for co-immunoprecipitation. Briefly, cells were lyzed with NP40 lysis buffer (Beyotime, Haimen, China) for co-immunoprecipitation. Followed by centrifugation, the supernatants were pre-cleared with 20 μL protein G agarose beads (Beyotime, Haimen, China) coupled with mouse or rabbit IgG for over 2 h; and then were exposed to 20 μL protein G agarose beads coupled with the indicated antibodies for over 6 h. The beads were washed 3 times with 1 mL PBS for 20 min each. The precipitants were dissolved with the SDS loading buffer for Western blotting analyses.

### Assays for proteasome activity

The activity of 26 S proteasome was determined as described previously [44]. Briefly, cells were harvested and washed with PBS (pH 7.4), pelleted by centrifugation and then lyzed in Lysis buffer (pH 7.5) [50 mM Hepes, 5 mM EDTA, 150 mM NaCl, and 1% Triton] on ice for 30 min. After that, whole cell lysates were centrifugated at 12000 ×g and 4°C for 10 min. The supernatants were aspirated. The reaction buffer (pH 8) contained 20 mM HEPES, 0.5 mM EDTA, and 0.035% SDS. Reaction mixtures in a total volume of 100 μL including reaction buffer (85 μL), cell extracts (5 μL), and the fluorogenic proteasome substrate Z-LLL-AMC (10 μL) (Calbiochem, La Jolla, CA) were incubated at 37°C for 1 h. For cell-free assays (the cell lysates were treated with triptolide), triptolide was added into the mixtures. For cellular assays (Cells were treated with triptolide and then subjected to lysis), the protein concentration was determined by the Micro BCA protocol (Pierce, Rockford, USA) and added the cell extracts with the same protein concentration into the mixtures. Cleavage activity was monitored continuously by detecting free 7-amido-4-methylcoumarin with a fluorescence plate reader (Gemini, Molecular Devices, USA) at 380/460 nm. As controls for drug studies, Z-LLL-AMC was incubated with drugs in Reaction buffer without cell extracts and measurements of proteasome activity were corrected when necessary.

### Luciferase activity assays

For luciferase activity assays, cells were co-transfected with 60 ng HRE luciferase reporter plasmid and renilla luciferase reporter vector pGL-3 per well using Lipofectamine™2000 for 24 h. Cells were treated with triptolide for further 12 h and harvested for the luciferase activity analyses using the dual luciferase reporter assay system (Beyotime, Haimen, China). Luminence was measured with GloMax^® ^96 Microplate LuminometerW/Dual injectors (Promega, Madison, USA). The firefly luciferase luminescence activity was normalized to the control renilla luciferase activity.

### Reverse transcription-PCR analyses

Cells were treated with triptolide for the indicated time. Total RNA was isolated with the Trizol reagent. Total RNA was reverse transcribed using Superscript™III reverse transcriptase and cDNA was used for PCR with the following primers (synthesized by Sanggon Corporation, Shanghai, China): β-actin, 5'-TGA CGG GGT CAC CCA CAC TGT GCC CAT CTA-3'(forward), 5'-CTA GAA GCA TTG CGG TCG ACG ATG GAG GG-3'(backward); and HIF-1α, 5'-CTC AAA GTC GGA CAG CCT CA-3'(forward), 5'-CCC TGC AGT AGG TTT CTG CT-3'(backward). Amplification was done for 35 cycles, each with denaturation at 94°C for 1 min, annealing at 55°C for 1 min and extension at 72°C for 1 min. The products were analyzed using agarose gel electrophoresis and visualized by ethidium bromide staining.

### Real time-PCR Analyses

Cells were lyzed with the Trizol reagent and total RNA was isolated with chloroform and isopropyl alcohol. One-microgram RNA was subjected to reverse transcription with the RT reagent kit (TaKaRa, Dalian, China) according to the manufacturer's instructions. Then the cDNA was amplified by Real-time PCR with the SYBR PrimeScript RT-PCR kit (Takara, Dalian, China) with the following primers (synthesized by Sanggon Corporation, Shanghai, China): β-actin, 5'-TGA CGG GGT CAC CCA CAC TGT GCC CAT CTA-3'(forward), 5'-CTA GAA GCA TTG CGG TCG ACG ATG GAG GG-3'(backward); HIF-1α, 5'-CTC AAA GTC GGA CAG CCT CA-3'(forward), 5'-CCC TGC AGT AGG TTT CTG CT-3'(backward); CAIX[31], 5'-CTT GGA AGA AAT CGC TGA GG-3'(forward), 5'-TGG AAG TAG CGG CTG AAG TC-3' (backward); BNIP3[31], 5'-TGC TGC TCT CTC ATT TGC TG-3' (forward), 5'-GAC TCC AGT TCT TCA TCA AAA GGT-3'(backward); and VEGF[31], 5'-CTA CCT CCA CCA TGC CAA GT-3'(forward), 5'-CCA CTT CGT GAT GAT TCT GC-3'(backward). The alteration of mRNA expression in cells treated with or without triptolide was assessed by delta delta C_t _method [45].

### ELISA assays for VEGF secretion

The amount of secreted VEGF was tested as described previously [46]. The medium was replaced with 1.5 mL/well of fresh medium, and the cells were subjected to hypoxia or normoxia in the presence or absence of triptolide at the indicated concentrations for 12 h. Then, the cell supernatants were collected, clarified by centrifugation at 1,000 g for 5 min, and stored at -20°C. VEGF in the supernatant was determined with a VEGF-ELISA kit according to the manufacturer's instructions (Jingmei Biotech Co, Beijing, China). Results were normalized to the cell number.

### Small interfering RNA (siRNA) transfection

siRNAs (HIF-1α siRNA-1 targeting 5'-CUG AUG ACC AGC AAC UUG ATT-3' [47], HIF-1α siRNA-2 targeting 5' -GCU CAA UUU AUG AAU AUU ATT-3', HIF-1α siRNA-3 targeting 5'-GAA GGA ACC UGA UGC UUU ATT-3' and GFP (scrambled) siRNA targeting 5'-GAC CCG CGC CGA GGU GAA GTT-3') were obtained from GenePharma (Shanghai, China). The transfection with siRNA was conducted with Lipofectamine RNAimax (Invitrogen, Carlsbad, CA) according to the manufacturer's instructions. After 24 h, the cells were treated with or without triptolide for the indicated time.

### Flow cytometry

SKOV-3 cells (2 × 10^5^) transfected with HIF-1α siRNA or GFP siRNA were seeded into 6-well plates overnight and then treated with or without triptolide for 36 h. Apoptosis was analyzed by flow cytometry [48]. The sub-G1 cells were considered as apoptotic cells.

### Rat aortic ring assays

Rat aortic ring assays were performed as described before [49]. The aortas were harvested from 6-week-old-aged Sprague-Dawley rats. Each aorta was cut into 1-mm slices and embedded in 30 μL Matrigel in 24-well plates. The aortic rings were then fed with 500 μL of M199 medium (10% FCS) with different concentrations of triptolide or the solvent control. After 5-d incubation at 37°C in a CO_2 _incubator, each aorta was photographed.

### Data Analyses

Data were presented as mean ± SD, and differences were considered significant when P < 0.05 as determined by Student's t test.

## Competing interests

The authors declare that they have no competing interests.

## Authors' contributions

ZLZ, ZGL, JD and ZHM designed the study; ZLZ, ZGL, BY, YJ, YC, JMF, MD and LJT performed experiments; ZLZ, JD and ZHM analyzed data; ZL and YCL extracted and purified triptolide, tested its purity and wrote the chemical section. ZLZ and ZHM wrote the paper. All authors read and approved the final manuscript.
